# Vasculogenic mimicry in hepatocellular carcinoma contributes to portal vein invasion

**DOI:** 10.18632/oncotarget.12867

**Published:** 2016-10-25

**Authors:** Chen Jue, Wu Zhifeng, Zhang Zhisheng, Cui Lin, Qian Yayun, Jin Feng, Gu Hao, Ishikawa Shintaro, Tadashi Hisamitsu, Guo Shiyu, Liu Yanqing

**Affiliations:** ^1^ Institution of Integrated Traditional Chinese and Western Medicine, Medical College, Yangzhou University, Yangzhou, Jiangsu, China; ^2^ Department of Oncology, The Second People's Hospital of Taizhou Affiliated to Yangzhou University, Taizhou, Jiangsu, China; ^3^ Department of Physiology, School of Medicine, Showa University, Tokyo, Japan; ^4^ Department of Oncology, Zhongshan Hospital Affiliated to Fudan University, Shanghai, China

**Keywords:** hepatocellular carcinoma (HCC), vasculogenic mimicry (VM), portal vein invasion (PVI), Notch1

## Abstract

Portal vein invasion (PVI) is common in hepatocellular carcinoma (HCC) and largely contributes to tumor recurrence after radical tumor resection or liver transplantation. Vasculogenic mimicry (VM) was an independent vascular system lined with tumor cells and associated with poor prognosis of HCC. The present study was conducted to evaluate the relationship between VM and portal vein invasion. A total of 44 HCC cases receiving anatomic liver resection were included in the study and were divided into groups with and without PVI. The prevalence of VM in each group was examined by CD34-PAS dual staining. The regulatory molecules of VM formation such as Notch1, Vimentin and matrix metalloproteinases (MMPs) were investigated by immunohistochemical staining. Analysis was performed to explore the association of PVI, VM and the VM regulatory molecules. PVI was found in 40.91% (18/44) cases and VM was found in 38.64% (17/44) cases in total samples. The incidence of VM was 72.22% (13/18) in PVI group while it was 15.38% (4/26) in non-PVI group (P<0.001), VM formation was positively correlated with PVI (r=0.574, P<0.001). The VM forming regulatory molecules such as Notch1, Vimentin, MMP-2 and MMP-9 were found to be correlated with PVI in HCC patients. Taken together, our results suggested that VM formation, alone with its regulatory molecules, is the promoting factor of PVI in hepatocellular carcinoma.

## INTRODUCTION

HCC is a common malignant tumor causing high cancer related mortality in East Asia, especially in China [1]. Hepatic resection and liver transplantation have been commonly applied in patients with early stage of the disease; and these approaches have largely improved the disease free survival (DFS) and overall survival (OS) in some HCC patients. However, intrahepatic recurrence and distant metastasis remain to be main factors accounting for the failure of treatments [2]. Therefore, it is urgent to delineate factors that promote the recurrence and metastasis of the disease and its underlying mechanisms.

Emerging evidences suggest that PVI occurs in early stage of HCC is associated with the metastasis and predicts poor prognosis [3–5]. HCC patients complicated with PVI will have a median survival time of less than 4 months if no effective interventions were taken promptly [5]. Recently, studies have demonstrated several risk or predicting factors for PVI, such as tumor size, grade of differentiation, capsule formation, serum level of alpha fetoprotein (AFP), alkaline phosphatase (ALP) and platelet counts, by using univariable or multivariable analysis [6]. However, few of them give a distinct definition for the relationship between the factors and PVI. Currently, knowledge on the risk factors and the corresponding underlying mechanisms that account for early presence of PVI are still lacking.

Vasculogenic mimicry (VM) was identified in melanoma at the end of last century and is accepted presently as a predictor for poor prognosis in numerous solid malignant tumors including HCC [7–12]. During tumor development, when the endothelia dependent angiogenesis from host is incapable of matching the need of nutrition and oxygen supplements for tumor growth, VM occurs and is in charge of the blood supply [13]. A classic VM channel is lined internally with high aggressive tumor cells and a layer of basal lamina rich in extracellular matrix (ECM) [14]. With the progression of cancer, VM gradually develops into another mosaic vessel, a vessel constructed with tumor cells combining with endothelial cells together, ultimately connects and merges with endothelial dependent vessels (EDV) [15]. Cumulating discoveries show that presence of VM could be considered as a metastatic route as well as a potential pro-metastasis factor [13].

Notch1 is a predominant receptor of Delta-like ligand and Notch (DLL-Notch) receptor family and contributes to cell fate decision, proliferation and differentiation. As an angiogenesis promoting molecule, Notch1 together with vascular endothelial growth factor A (VEGFA) manipulate sprouting angiogenesis and guarantee the vessels functions [16]. In addition, Notch1 also functions as an oncogene in a number of malignancies including HCC [17–19]. There are accumulating evidences indicating the association between Notch and VM formation in HCC [20, 21]. Our previous results (manuscript in press) proved that increased Notch1 was associated with VM development in HCC, and overexpression of Notch1 in HepG2 cells could endow the cells with the VM forming ability. Underlying mechanism was that overexpression of Notch1 probably induced epithelial to mesenchymal transition (EMT).

Because of the marked vascularity inside HCC, regional portal vein and its branches turn into the main countercurrent vessel for tumor mass [22]. Tumor related angiogenesis thus potentially contributes to PVI. A study has demonstrated that microvascular density and VEGF have close association with PVI [23]. However, the relationship between VM and PVI is still unclear. We speculated that VM may contribute to the incidence of PVI. The present study was conducted to investigate the relationship between VM and PVI in patients with HCC.

## RESULTS

### Demographics and clinical characteristics of HCC patients with and without PVI

A total of 44 HCC patients receiving anatomical resection in our hospital was enrolled in the present study and among them, 18 (40.91%) cases were identified with PVI by postoperation pathological examination. To determinate whether demographics and clinical characteristics contributed to PVI, all the patients were divided into two groups, one with PVI and the other without PVI (PVI vs. non-PVI). The demographics and clinical characteristics of these two groups were examined and evaluated using t-test or Chi-square test (Table 1). The majority of these baseline variables showed no significant differences between the two groups except AFP. Statistical result showed a significant increase of AFP in PVI group compared to non-PVI group (P<0.05). As for the etiology, a total of 30 patients (68.18%) were found to be HBV carriers and HBV-DNA copies increased in PVI group, but there was no significant difference between the PVI and non-PVI group (P>0.05). This finding was not surprising because HBV is still the main cause of hepatitis and HCC in China. Among patients with and without PVI, there were significant differences in tumor diameters and Edmonson Steiner grade (P<0.05 and 0.05 respectively), while there were no statistical significances in the tumor marginal state and liver cirrhosis complication (P>0.05 and 0.05 respectively).

**Table 1:** Demographics and clinical characteristics of HCC patients with and without PVI

Characteristics		All (n=44)	PVI (n=18)	Non-PVI (n=26)	*P* value
Age		58.52±8.13	58.67±8.76	58.42±7.83	0.924
Gender	Male	38 (86.36%)	16 (88.89%)	22 (84.62%)	1.000
	Female	6 (13.64%)	2 (11.11%)	4 (15.38%)	
Alcoholism	Yes	9 (20.45%)	3 (16.67%)	6 (23.08%)	0.716
	No	35 (79.55%)	15 (83.33%)	20 (76.92%)	
HBV-DNA	≥1000cps/ml	30 (68.18%)	15 (83.33%)	15 (57.69%)	0.104
	<1000cps/ml	14 (31.82%)	3 (16.67%)	11(42.31%)	
HCV^*^		N/A	N/A	N/A	-^#^
ALT (IU/L)		48.89±6.28	56.16±45.32	43.85±44.08	0.373
TBIL (μmol/ml)		15.73±6.28	16.51±5.11	15.20±7.02	0.504
Child-Pugh classification	A	37 (84.09%)	14 (77.78%)	23 (88.46%)	0.419
	B	7 (15.91%)	4 (22.22%)	3 (11.54%)	
AFP	≥400 ng/ml	34 (77.27%)	17 (94.44%)	17 (65.38%)	0.031^*^
	<400 ng/ml	10 (22.73%)	1 (5.56%)	9 (34.62%)	
ECOG Scores	0-1	34 (77.27%)	12 (66.67%)	22 (84.62%)	0.273
	2	10 (22.73%)	6 (33.33%)	4 (15.38%)	
Resection margin	R0	40 (90.91%)	17 (94.44%)	23 (88.46%)	0.634
	R1	4 (9.09%)	1 (5.56%)	3 (11.54%)	
Cirrhosis	Yes	35 (79.55%)	15 (83.33%)	20 (76.92%)	0.716
	No	9 (20.45%)	3 (16.67%)	6 (23.08%)	
Tumor diameters		6.84±2.85	8.06±2.14	6.00±3.01	0.017^*^
Edmonson-Steiner grade	I-II	37 (84.09%)	12 (66.67%)	25 (96.15%)	0.013^*^
	III-IV	7 (15.91%)	6 (33.33%)	1 (3.85%)	

SD, standard deviation; HBV, hepatitis B virus; HCV, hepatitis C virus; ALT, alanine aminotransferase; AFP, Alpha-fetoprotein; TBIL, total bilirubin; N/A, not available.

# missing data.

* P<0.05.

### Prevalence of VM in HCC patients with and without PVI

Since VM has no endothelial cells and is consisted of tumor cells with basement membrane components, it is not stained by CD34 but stained positively by PAS. Therefore, net-like structures with PAS positive and CD34 negative staining were considered as positive VM formation in tumor tissues. In this study, 17 (33.64%, 17/44) cases with VM were found. Among the clinical characteristics summarized in Table 2, except for Edmonson-Steiner grade (r=0.421, P<0.01, Figure 1 and Table 2), none of them were found to be associated with VM formation. Positive VM structures were found both in PVI and non-PVI group. There were 13 (72.22%, 13/18) cases with VM in PVI group while only 4 (15.38%, 4/26) cases in non-PVI group (P<0.001, Figure 1). The linear regression analysis further confirmed that VM formation had a positive correlation with PVI (r=0.574, P<0.001, Figure 1).

**Table 2:** Correlations between VM and clinical characteristics

Variables	Correlation coefficient	P value
Age	0.044	0.776
Gender	0.093	0.549
Alcoholism	0.119	0.443
HBV-DNA	0.141	0.361
HCV^*^	-	-
ALT (IU/L)	0.226	0.140
TBIL (μmol/ml)	0.1242	0.359
Child-Pugh classification	0.176	0.253
AFP	0.010	0.948
ECOG Scores	0.015	0.922
Resection margin	0.021	0.873
Cirrhosis	0.055	0.722
Tumor diameters	0.204	0.184
Edmonson-Steiner grade	0.421	0.004^#^

* missing data

# significant difference.

**Figure 1:**
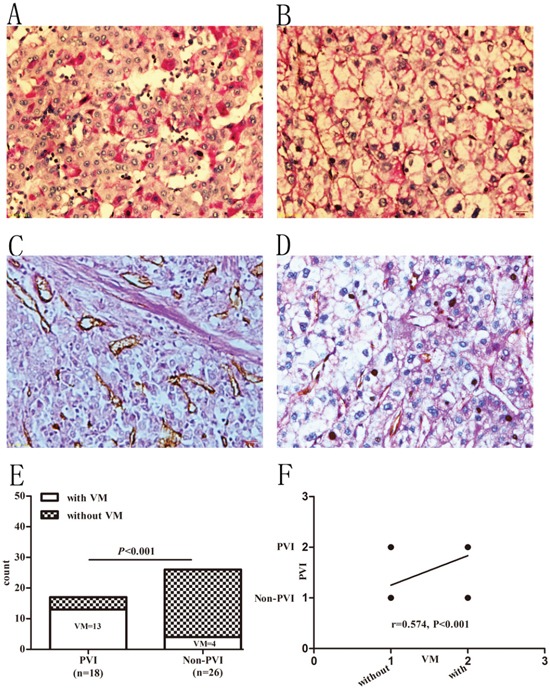
Prevalence of VM in HCC patients with and without PVI **A** and **B.** PAS staining in HCC specimens. A: PAS staining in well to moderate differentiated samples. B: PAS staining in poorly differentiated samples. **C** and **D.** PAS-CD34 double-staining. C: specimen without VM. D: specimen with VM. Original magnification: 400×. **E.** Prevalence of VM in PVI and non-PVI groups. **F.** Presence of VM positively associated with PVI.

### Notch1 associated with VM formation and PVI

**Figure 2:**
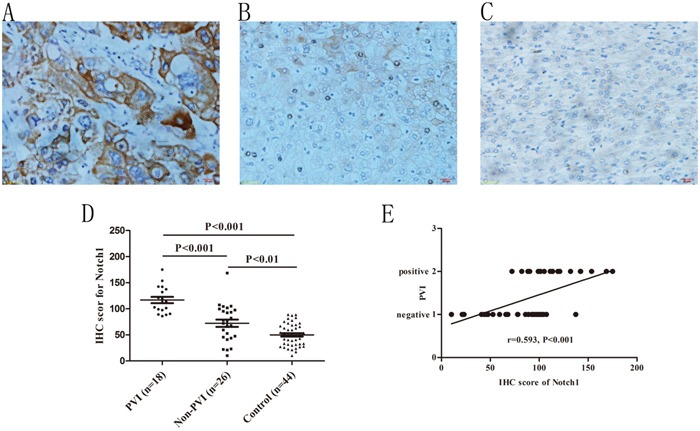
Immunohistochemical staining of Notch1 in HCC specimens and its association with PVI **A.** Notch1 staining in HCC specimens with PVI, **B.** Notch1 staining in HCC specimens without PVI, **C.** Notch1 staining in adjacent non-tumor specimens. Original magnification: 400×. **D.** Different staining density and proportion of Notch1 in specimens of PVI, non-PVI and control (non-tumor) specimens. **E.** Spearman correlation analysis indicated a positive correlation between Notch1 expression and PVI.

Some researchers suggested that Notch1 played important roles in VM formation and correlated with poor prognosis in HCC [20]. Our previous results showed that Notch1 expression has a positive correlation with VM in HCC (r=0.590, P<0.001) and artificial overexpression of Notch1 in HCC cells could promote VM (manuscript in press). To evaluate whether Notch1 expression correlated with PVI, we compared it among HCC patients with and without PVI. As shown in Figure 2, both in PVI and non-PVI groups, IHC scores for Notch1 (116.81±25.57 and 72.22±35.53) were found increased compared to corresponding adjacent non-HCC tissues (49.99±21.63, P<0.001 and 0.001 respectively). In addition, PVI group had a higher Notch1 score than non-PVI group (P<0.01, Figure 2). Correlation analysis indicated that Notch1 expression had a close association with PVI (r=0.593, P<0.001, Figure 2). Combining with our previous findings, the results suggested that expression of Notch1 is associated with VM formation and PVI.

### Positive vimentin staining in HCC cells associated with PVI

**Figure 3:**
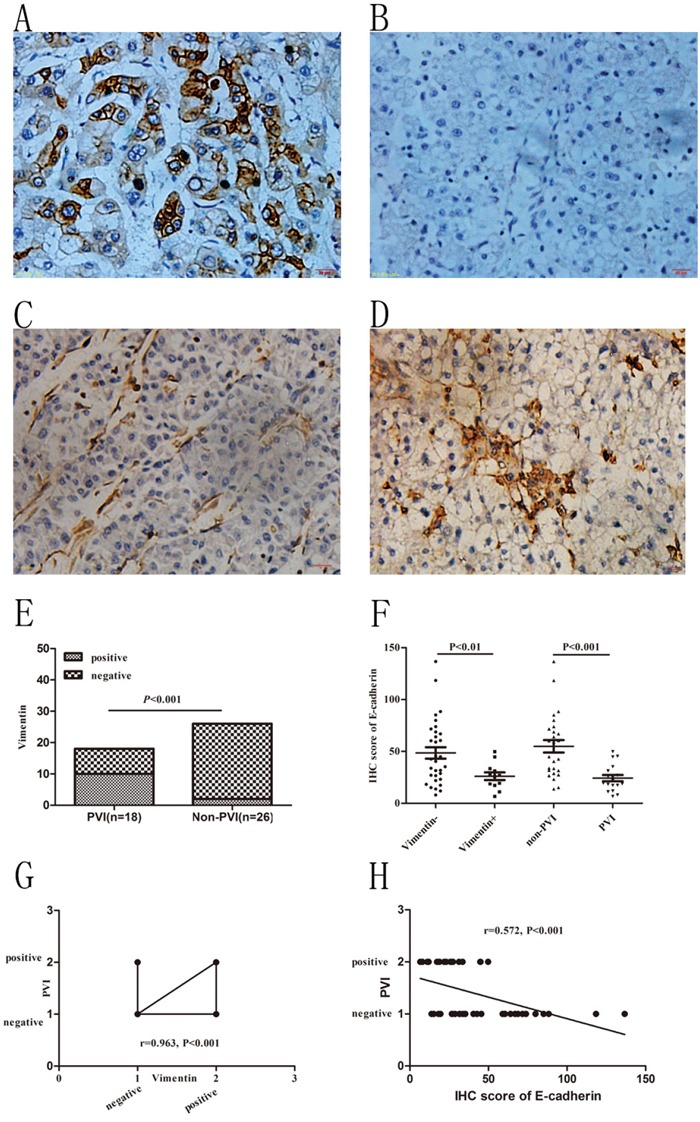
Immunohistochemical staining of Vimentin in HCC specimens and its association with VM and PVI **A** and **B.** E-cadherin staining in non-PVI and PVI samples. **C** and **D.** Negative and positive Vimentin staining in non-PVI and PVI samples. Original magnification was 400×. **E.** Incidence of positive vimentin staining in PVI and non-PVI patients. Chi-Square test was used. **F.** E-cadherin staining in Vimentin positive and negative samples; E-cadherin staining in non-PVI and PVI samples. T-test was used. **G.** Increased Vimentin positively associated with PVI. Spearman correlation was used. **H.** Decreased E-cadherin negatively associated with PVI. Spearman Correlation analysis was used.

Vimentin was normally expressed in the stroma and microvascular wall of HCC but not in HCC cells. A positive staining of Vimentin in HCC cells tended to reflect epithelial to mensenchymal transition (EMT) which was suggested to contribute to VM formation [24]. In this study we found 12 (27.27%, 12/44) specimens with positive Vimentin staining in the cytoplasm of HCC cells. As for its counterpart, E-cadherin was found decreased in the 12 cases compare to the rest 32 cases (26.09±12.70 vs. 48.51±31.15, P<0.01, Figure 3). Correlation analysis confirmed that Vimentin was positively correlated with VM formation (r=0.963, P<0.001) while E-cadherin was negatively correlated with VM (r= −0.527, P<0.001). In our previous study we found that overexpression or knockdown expression of Notch1 in HCC cells could increase or decrease Vimentin expression (manuscript in press). In accordance with this finding, we found in this study that increased Vimentin and decreased E-cadherin were positively and negatively correlated with Notch1 expression respectively (r=0.561, P<0.001; r= −0.524, P<0.001). In PVI group we found 10 (55.56%, 10/18) cases with positive Vimentin staining while only 2 (7.69%, 2/26) cases in non-PVI group (P<0.001, Figure 3). E-cadherin was found significantly declined in PVI group compared with non-PVI (24.30±12.83 vs. 54.92±30.64, P<0.001, Figure 3). Liner regression analysis confirmed that increasing Vimentin and decreasing E-cadherin were closely associated with PVI (r=0.582, P<0.001; r= −0.572, P<0.001, respectively. Figure 3).

### Increase of MMPs associated with VM formation and indicated higher incidence of PVI

**Figure 4:**
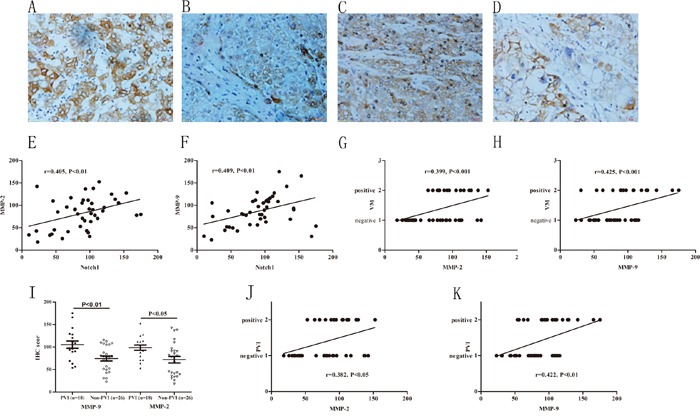
Increased expressions of MMPs associated with VM and higher incidence of PVI **A** and **B.** MMP-9 immunohistochemical staining in HCC with and without PVI, original magnification: 400×. **C** and **D.** MMP-2 immunohistochemical staining in HCC with and without PVI, original magnification: 400×. **E** and **F.** MMP-2, MMP-9 IHC score were correlated with Notch1. **G** and **H.** MMP-2 and MMP-9 expression positively correlated with VM. I: MMP-2 and MMP-9 significantly increased in PVI group compared with non-PVI group. J and K: MMP-2, MMP-9 IHC score were positively correlated with PVI.

Emerging evidences have suggested that Notch1 induces increase of MMP-2 and MMP-9 expression that were important in cancer invasion and the extracellular matrix remolding during VM development [25, 26]. In this study, we found that expression of MMP-2 and MMP-9 were positively correlated with Notch1 expression (r=0.405, P<0.01; r=0.409, P<0.01 respectively, Figure 4). Furthermore, the increased expressions of MMP-2 and MMP-9 were positively associated with VM formation (r=0.425, P<0.001; r=0.399, P<0.01 respectively, Figure 4). In patients with PVI, IHC scores of MMP-2 and MMP-9 were 98.63±24.89 and 105.21±34.48 respectively, while in patients without PVI, the scores were 71.94±37.14 and 74.42±27.87 respectively. The expressions of MMP-2 and MMP-9 have significant difference between the PVI and non-PVI groups (P<0.01 and 0.01 respectively, Figure 4). Further correlation analysis indicated that MMP-2 and MMP-9 have close associations with PVI (r=0.0.382, P<0.05; r=0.422, P<0.01 respectively, Figure 4). These results suggested that overexpression of MMP-2 and MMP-9 is associated with VM formation and PVI.

## DISCUSSION

A number of studies have demonstrated that PVI exists in 44%-62.2% of early and mid-stage HCC patients who receive hepatic resection or liver transplantation [22]. Occurrence of PVI indicates a poor prognosis [4]. Cancer staging and postoperative treatment varied in patients with or without PVI. Currently, clinical trials have been conducted to validate various techniques for example surgical resection, transcatheter arterial chemoembolization (TACE), hepatic arterial infusion chemotherapy, radioembolization and sorafenib for the treatment of HCC [27–29]. Besides, attention has been paid to understand the promoting factors of PVI and corresponding mechanisms [22]. Our current study contributed to elucidate the role of angiogenesis related molecules such as Notch1, Vimentin, MMP-2 and MMP-9 in promoting VM formation and PVI in HCC. Evidence provided in this study supports the hypothesis that VM, as a pathological auto circulation in the tumor mass, may induce PVI in HCC.

The interaction between tumor internal circulation and the host blood supply are critical in malignancies that depend on hematogenous spread such as HCC. VM channel provides a pathway and functions as an important promoter in early tumor invasion and metastasis. VM normally exists in the early phase of tumor expansion and supplies the nutrition and oxygen together with mosaic vessel and endothelial dependent vessel and thus provides a chance for tumor cells to be exposed directly to blood flow [30]. On the other hand, cells lining VM channels normally exhibit poor pathological characteristics such as high invasiveness, plasticity, self-differentiation and potentiality of ECM [31]. These features have been suggested to be associated with tumor metastasis.

Currently, the precise mechanisms related to PVI development are still contentious. Not only the aberrant vascular structures inside tumor mass but also the malignant phenotype of tumor cells and microenvironment in local portal vein are all contributors of PVI. In this study, we demonstrated that VM participates in tumor blood supply via connecting to endothelia-derived-vessels (EDV) originated from the host. This pathological vasoganglion not only promotes vascular abnormality, decreases blood supply resistance, subsequently leads to the hypertension of local vein; but also provides a shortcut for tumor cells to escape from the origin and migrate to the distance. In addition, with the development of VM, tumor cells obtain more malignant characteristics such as deattachment, migration, invasion and the ability to degrade and remold extracellular matrix (ECM), these factors are the co-promotors of PVI [22].

It has been documented that angiogenesis related molecules such as VEGFR1, Notch, Twist, ZEB1, Snail and Slug are involved in VM formation [30]. Among these molecules, Notch is extensively studied in various malignancies for its roles in proliferation, differentiation, invasion and metastasis. A previous report demonstrates that Notch1 is involved in VM formation in HCC and suppressing Notch1 inhibits the progression of VM [20]. A recent study confirmed that Notch participated in VM by manipulating NODAL expression [32]. Our previous study shows that increased Notch1 is associated with VM formation in HCC. Increased Notch1 induced by TGF-β1 or lentivirus mediated overexpression can promote HCC cell forming VM both *in vitro* and *in vivo* (manuscript in press). In the present study, Notch1 expression is higher in PVI group compared with non-PVI group. Increased Notch1 has a close association with PVI. Combining with our previous results, it is rational to consider that Notch1 promotes VM formation in HCC and subsequently induces the occurrence of PVI.

**Figure 5:**
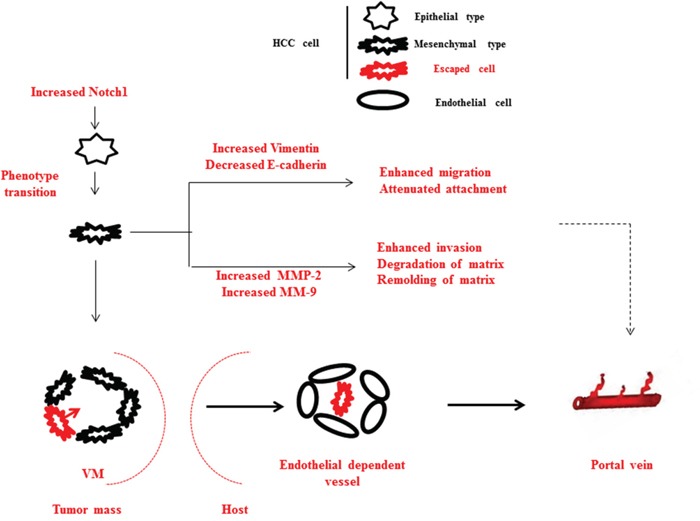
Schematic diagram of summary of this study Elevated Notch1 induces a cohort of tumor cells translate from epithelial to mesenchymal phenotype in HCC. During the process, Vimentin increases and E-cadherin decreases in cells with malignant phenotype, therefore migration and deattachment are enhanced; MMP-2 and MMP-9 increases, therefore ECM degradation and remolding are enhanced. These support the PVI development at the cellular level. Additionally, EMT induced VM aggravates vasoganglion abnormality inside tumor mass, connects portal vein and internal circulation of tumor mass, therefore offers pathways for tumor cells to metastasize into portal vein. These support the PVI development at the circulation level.

Existing data have shown a positive impact of EMT and ECM degradation on VM channel formation [33]. During EMT process, Vimentin is predominantly expressed while E-cadherin is suppressed. As a result, vimentin and E-cadherin were used as important markers for EMT in studies related to VM formation [34]. Alteratively, acquisition of mesenchymal phenotype, especially endothelial phenotype indicates the acquisition of potentiality for angiogenesis. In addition, decrease of E-cadherin and increase of Vimentin allow tumor cells easily to escape from the origin and metastasize. During degradation of ECM, MMPs especially MMP-2 and MMP-9 contribute to the proteolysis of the ECM whose degradation products together with tumor cells take part in the VM formation [35]. Although the roles of EMT and ECM degradation in VM formation have been well documented, the association between the two episodes and PVI was hardly proved. As shown in Figure 5, EMT promotes VM formation thus offers structure base for PVI. Simultaneously, changes of Vimentin and E-cadherin, increase of MMPs lead HCC cells easily to enter into portal vein and subsequently degrade matrix and seed on the vessel wall. Collectively, these findings support the hypothesis that VM formation is associated with PVI.

This study has some limitations. It was reported that HBV infection can promote PVI via activating TGF-β signaling [22]. However we fail to find the significant differences of HBV infection and hepatic fibrosis between PVI and non-PVI group. Further analysis based on larger sample size and broader mechanism exploration depended on cellular and molecular biological experiments should be performed. Although we proved that VM connects to dismal branches of portal vein via EDV by pathological staining methods and statistics, corresponding ultrastructure and mechanisms are still unclear. In our previous study we have demonstrated that Notch1 is an upstream factor that regulate the expression of Vimentin and E-cadherin, MMP-9 and MMP-2. However, in the current study, we are still unable to explain how does Notch1 decide or impact the HCC cells to complete the terminal seeding and transplantation in portal vein. A larger cohort observation and some complex mechanism studies should be performed. AFP was found different between PVI and non-PVI group. We consider this may be associated with potentially subclinical tumor activation.

In summary, our results demonstrated that VM formation is positively correlated with PVI in HCC. Overexpression of Notch1, followed by the changes of expression of Vimentin, E-cadherin and MMPs are closely associated with PVI in HCC. These findings will help prognostic estimation and provide treatment target for HCC patients with PVI.

## MATERIALS AND METHODS

### Patients and HCC specimens

Previous study suggested that incidence of PVI in HCC patients receiving resection is approximately 46.8% [36]. According to the reported incidence, we estimated the sample size with the 80% statistical power and the result indicated the sample size should be more than 30 cases. As a result, a total of 44 patients who were diagnosed with HCC and received anatomic liver resection in the Second People's Hospital of Taizhou (Jiangsu, China) between 2009 and 2015 were enrolled in the study. The post-operative histopathologic diagnosis for HCC and PVI were independently confirmed by two pathologists through routine histopathological examination based on H&E staining. Individuals with mixed hepatocellular carcinoma and cholangiocarcinoma were excluded. None of the 44 patients received any preoperative anti-cancer treatments. The demographic and clinical characteristics were collected from the electronic medical record system. All specimens were fixed in formalin and embedded in paraffin. This study was reviewed and approved by the institutional ethic committee of the Second People's Hospital of Taizhou. All patients were requested to sign the informed consent for participation. The characteristics of patients were summarized in Table 1.

### Immunohistochemical staining

Immunohistochemical staining was performed as previously described [37]. Briefly, 4μm thick sections were routinely dewaxed and rehydrated. Sections were then heated at 100°C in citrate buffer (0.01 M, pH 6.0) for 10 min using a microwave oven for antigen retrieval. After that, sections were treated with 3% H_2_O_2_ for 20 min to inactivate the endogenous peroxidase and incubated with 10% goat serum for 30 min to block the nonspecific binding. Next, sections were incubated with primary antibodies at 4°C overnight. The list of antibodies used was summarized in Table 3. After rinsing, sections were incubated with biotin labeled secondary antibody against responding primary antibodies for 30 min at 37°C. Then sections were rinsed and incubated with 3,3′-diaminobenzidinetetrahydrochloride (DAB) for 10 min at room temperature. Slides were then counterstained with hematoxylin. Negative control was achieved by replacing the primary antibodies with PBS.

**Table 3:** Primary antibodies used in immunohistochemical staining

Primary antibodies	Serial Number	Dilution	Source	Company
Notch1	4380	1:100	Rabbit	CST, Danforth, USA
MMP-2	Ab7033	1:200	Mouse	Abcam, Camb, UK
MMP-9	Ab73734	1:200	Rabbit	Abcam, Camb, UK
E-cadherin	3195	1:150	Rabbit	CST, Danforth, USA
Vimentin	5741	1:100	Rabbit	CST, Danforth, USA
CD34	3569	1:50	Mouse	CST, Danforth, USA

CST, cell signaling technology.

### PAS-CD34 dual staining

The staining procedure was performed as previous description [38]. Briefly, immunohistochemical staining was applied to perform CD34 staining. The procedure was the same as the description above (CD34 primary antibody dilution was 1:200). After immunihistochemical staining, sections were treated with 0.5% periodic acid solution for 10 min and rinsed with distilled water for 5 min. In a dark chamber, sections were treated with Schiff solution for 15-30 min. After rinsing with distilled water, sections were counterstained with hematoxylin.

### Staining evaluation

All the slides were evaluated by two independent pathologists who were blinded with the study background and outcomes. For immunohistochemical staining of Notch1, E-cadherin, MMP-9 and MMP-2, the slides were imaged digitally with the same light exposure and evaluated by Image Pro Plus (IPP), a digitalized immunohistochemistry scoring program (Media Cybernetics, San Diego, CA). For immunohistochemical staining of Vimentin, because the target staining location was in cytoplasm or cell membrane other than microvascular wall, the staining located in HCC cells agreed by two pathologists was considered positive staining; conversely, it was considered negative staining if the staining was in microvascular wall.

### Statistical analysis

Comparisons between PVI and non-PVI groups were performed using the Student's t-test for continuous variables according to normal distribution and Chi-square test for categorical variables. Pearson and Spearman correlation coefficient were used to determine the relationship between two variables. P values less than 0.05 were considered statistically significant. All analysis was performed with SPSS software version 22.0 for Windows (SPSS, Chicago, IL).
